# A Polymorphism in the *BDNF* Gene (rs11030101) is Associated With Negative Symptoms in Chinese Han Patients With Schizophrenia

**DOI:** 10.3389/fgene.2022.849227

**Published:** 2022-03-16

**Authors:** Junjiao Ping, Jie Zhang, Jing Wan, Caiying Huang, Jiali Luo, Baoguo Du, Tingyun Jiang

**Affiliations:** ^1^ Department of Psychiatry, The Third People’s Hospital of Zhongshan City, Zhongshan, China; ^2^ Joint Laboratory of Psychiatric Genetic Research, The Third People’s Hospital of Zhongshan, Zhongshan, China; ^3^ Department of Psychiatry, Gannan Medical University, Ganzhou, China; ^4^ Department of Early Intervention, Third People’s Hospital of Zhongshan City, Zhongshan, China; ^5^ Department of Clinical Psychology, The Third People’s Hospital of Zhongshan City, Zhongshan, China

**Keywords:** schizophrenia, brain derived growth factor, negative symptoms, polymorphism (genetics), CREB (cAMP response element binding protein)

## Abstract

**Objective:** This study aimed to investigate the association between brain-derived neurotrophic factor (*BDNF*) and cyclic adenosine monophosphate response element binding protein (*CREB*) gene polymorphisms and schizophrenia.

**Methods:** This study used a case-control design, and diagnoses were made based on the Diagnostic and Statistical Manual of Mental Disorders-Fifth Edition criteria. One hundred and thirty-four patients with schizophrenia were recruited from the Third People’s Hospital of Zhongshan City from January 2018 to April 2020. Sixty-four healthy controls were recruited from the same region. Genotypes at the *BDNF* gene single nucleotide polymorphisms rs11030101, rs2030324, and rs6265 and the *CREB* gene single nucleotide polymorphisms rs6740584 and rs2551640 were determined using a MassARRAY mass spectrometer. Linkage disequilibrium and haplotype analyses were performed, and genotype and allele frequencies were compared between groups. The positive and negative symptom scale (PANSS) was used to evaluate the association between the *BDNF* and *CREB* gene polymorphisms and schizophrenic symptoms.

**Results:** There was no significant difference in genotype or allele frequencies for rs11030101, rs2030324, rs6265, rs6740584, or rs2551640 between schizophrenic patients and controls (p > 0.05). In addition, there were no significant differences in rs11030101, rs2030324, rs6265, rs6740584, or rs2551640 genotype frequencies between the two groups in the dominant, recessive, or over-dominant models (p > 0.05). Three loci in the BDNF gene and two loci in the CREB gene were in a state of strong linkage disequilibrium. The frequency of haplotype AAC (rs11030101/rs2030324/rs626), composed of three loci in the BDNF gene, was significantly increased in schizophrenic patients compared with control subjects. There were significant differences in the subscores of PANSSS for negative symptoms, in patients with different rs11030101 genotypes of the *BDNF* gene (*p* < 0.05). There was also significant differences in the PANSS scores for the general symptom G12 (judgment and lack of insight) in patients with different rs6265 genotypes of the *BDNF* gene (*p* < 0.05).

**Conclusion:** The *BDNF* gene rs11030101/rs2030324/rs6265 AAC haplotype was potentially associated with an increased risk of schizophrenia. In addition, genotypes at the rs11030101 and rs6265 loci may affect the negative symptoms and general symptoms of schizophrenic patients, respectively.

## Introduction

Schizophrenia is a serious mental illness. The course of the disease is often prolonged, the global prevalence rate is approximately 1%, and approximately half of patients with schizophrenia eventually develop a mental disability, which imparts a heavy burden on society, the family, and thepatient. The clinical symptoms of schizophrenia are complex. Patients generally have either i) no disturbance of intelligence or consciousness, ii) mainly positive symptoms, iii) negative symptoms, iv) cognitive impairment of the three core symptoms and emotional symptoms, or iv) other symptoms as the main symptom types (Duan et al., 2010; Müller, 2014; McMeekin et al., 2016; Lewis, 2018). A large number of clinical and basic research studies have shown that genetic factors play an important role in the pathogenesis of schizophrenia (Giegling et al., 2017). In recent years, association and linkage analysis studies have also confirmed that schizophrenia is a complex polygenic disease (Karl and Arnold, 2014). Schizophrenia is thought to be a brain disease characterized by neurodevelopmental disorders, leading to minor pathological changes in the brain. Meanwhile, the interaction of genetic, biological, and environmental factors plays an important role in the pathogenesis of schizophrenia (Flint and Munafò, 2014).

The product of the brain-derived neurotrophic factor (*BDNF*) gene is widely distributed in the central nervous system. It is a protein that promotes neuronal differentiation, growth, and development, and it plays an important role in maintaining normal brain function (Wang et al., 2015). *BDNF* is located on human chromosome 11p13 and contains 11 exons, with a total length of 70 kb. The rs11030101, rs2030324, and rs6265 loci in *BDNF* play an important role in the occurrence and development of schizophrenia. rs11030101 is located in an intron of the *BDNF* gene and plays an important role in the regulation of gene expression. rs2030324, also known as C270T, is located in the promoter region of the *BDNF* gene, and it affects BDNF expression (Ma et al., 2012). rs6265, also known as G196A (Val66Met), is a single nucleotide substitution at position 196 of the *BDNF* gene, resulting in the conversion of valine (Val) to methionine (Met) at codon 66. This variant reduces the secretion of BDNF by inhibiting its entry into secretory granules, thus affecting BDNF function (Ribasés et al., 2005). At present, there is no consistent conclusion on whether the rs11030101, rs2030324, and rs6265 loci of the *BDNF* gene are susceptibility genes for schizophrenia (Nieto et al., 2013; Zhang et al., 2016a).

BDNF is the most abundant neurotrophic factor in the human body. It activates intracellular signaling; induces the phosphorylation of cyclic adenosine monophosphate response element binding protein (CREB); activates related pathways; and, finally, activates CREB after binding to the related kinase tropomyosin receptor kinase B (Vyssotski et al., 2002). The *CREB* gene, located on chromosome 2q34, is a member of a family of active transcription factors. It is considered to be a potential regulator of the overall survival program of neurons, and it plays a key role in the central nervous system. Some studies have shown that CREB is dysfunctional in patients with schizophrenia. Ren et al. (2014) studied the expression and function of CREB protein and mRNA in the prefrontal cortex and cingulate gyrus. They found that the expression and activity of CREB protein and mRNA in the cingulate gyrus were lower in schizophrenic patients than in control subjects, suggesting that CREB abnormalities in the cingulate gyrus may be related to schizophrenia. It has been speculated that *CREB* gene polymorphisms may be involved in abnormal CREB function and may be related to schizophrenia (Ren et al., 2014; Forero et al., 2016).

Thus, the purpose of this study was to explore the associations between the *BDNF* gene polymorphisms rs11030101, rs2030324, and rs6265 and the *CREB* gene polymorphisms rs6740584 and rs2551640 and schizophrenia.

## Subjects and Methods

### Subjects

Patients with schizophrenia admitted to the Third People’s Hospital of Zhongshan City from 2018 to 2020 were recruited. They were included in the study if they: i) met the diagnostic criteria for schizophrenia, as recommended in the Diagnostic and Statistical Manual of Mental Disorders, Fifth Edition and were diagnosed jointly by two attending physicians who had received consistent training; ii) were aged 16–60 years; and iii) were of Han ethnicity. Patients with major somatic diseases (e.g., diabetes, hypertension, or cancer), nervous system diseases, or other mental disorders were excluded.

The control group was recruited from workers, nurses, volunteers, and patients undergoing health check-ups at the Third People’s Hospital of Zhongshan City. Subjects were included in the control group if they: i) had no history of mental illness after psychiatric screening; ii) had no family history of mental illness; iii) passed a physical examination; iv) were of Han ethnicity; and v) were aged 18–60 years. Individuals who were adopted or from single-parent families with an unknown family history or had major somatic diseases, such as diabetes, hypertension, or cancer, were excluded from participating in the control group.

This study was approved by the Ethics Committee of Zhongshan Third People’s Hospital, and either patients or their guardians provided informed consent.

### Methods

#### General Data and Clinical Information Collection

General demographic data, including age, sex, and educational level, were collected from all subjects. The clinical information of patients with schizophrenia, such as the age of onset, was also collected.

#### Psychotic Symptom Assessment

A medical history was obtained from patients with schizophrenia, and a psychiatric examination was performed on the same day. The positive and negative symptom scale (PANSS) was used to evaluate the psychotic symptoms of the patients. This scale includes a positive symptom score, negative symptom score, and general psychopathological symptom score. Patients were evaluated independently by two attending physicians who had received consistent training. The consistency between evaluators was high (r > 0.80).

#### Genomic DNA Extraction

Peripheral venous blood was collected into an ethylenediaminetetraacetic acid-containing vacuum anticoagulant tube and centrifuged at 3,300 rpm at room temperature. The leukocytes in the middle layer were collected and stored in a cryopreservation tube at −80°C for subsequent use. A TIANamp Genomic DNA Kit (centrifugal column type; DP304; Tiangen, Beijing, China) was used to extract genomic DNA, and the optical density was determined using a spectrophotometer (Thermo Fisher Scientific, Waltham, MA, United States). Genomic DNA extraction results were evaluated by gel electrophoresis.

#### Primer Synthesis

The MassARRAY single nucleotide polymorphism (SNP) genotyping platform (Agena Bioscience, San Diego, CA, United States) was used to genotype the *BDNF* and *CREB* gene SNPs. The Agena primer design tool (http://agenacx.com/) was used to design primers for the rs11030101, rs2030324, rs6265, rs6740584, and rs2551640 loci. The polymerase chain reaction (PCR) primers were diluted to 100 μM, and the PCR primer mixture was prepared according to the 1EXT 200 protocol. The extension primer mixture was prepared using 1:25 dilutions of the primers. After the configuration of the extension primer mixture was finalized, 2 μl of each primer were diluted 1:25 for mass spectrometric analysis. The ratio of the extension primers of individual loci was adjusted according to the test results.

#### PCR Amplification

A 1.5-ml Eppendorf tube was used to prepare a PCR master mix with a total volume of 4.1 μl, consisting of 1.850 μl of water, 0.625 μl of PCR buffer with 15 mM MgCl_2_, 0.325 μl of MgCl_2_, 0.100 μl of deoxy-ribonucleoside triphosphate (dNTP) mix, 0.1000 μl of primer mix, and 0.200 μl of HotStarTaq (Qiagen, Hilden, Germany). An 8- or 12-channel pipette was used to add 4 μl of master mix into each well of a 384-well plate, after which 1 μl of genomic DNA (20 ng/μl) was added. The samples were then mixed and centrifuged at 1,000 rpm for 1 min. The 384-well plates were carefully covered with a sealing film to prevent evaporation during the PCR procedure. The PCR amplification procedure was as follows: 94°C for 5 min; 45 cycles of 94°C for 20 s, 56°C for 30 s, and 72°C for 1 min; 72°C for 3 min; and hold at 4°C.

#### Alkaline Phosphatase Treatment

The PCR products were treated with shrimp alkaline phosphatase (SAP) to remove the free dNTPs in the system. An alkaline phosphatase reaction mix (1.53 μl of water, 0.17 μl of SAP buffer, and 0.3 μl of SAP) was prepared in a 1.5-ml Eppendorf tube, and 2 μl of the reaction mix were added to each well of a 384-well PCR plate containing 5 μl of PCR product. The SAP reaction was performed in a PCR machine using the following steps: 37°C for 20 min, 85°C for 5 min, and hold at 4°C.

#### Single-Base Extension Reaction

After alkaline phosphatase treatment, a single-base extension reaction was performed, as previously described by our research team (Ping et al., 2021) and Shah et al. (Shah et al., 2020)

#### Resin Purification and Chip Sampling

After PCR, Na^+^, Mg^2+^, K^+^, and other salt ions were removed using a cation exchange resin to avoid too many peaks in the analysis spectrum produced by the mass spectrometer, which would affect the analysis. Using a MassARRAY nanodispenser, a microvolume of sample was loaded onto the SpectroCHIP to prepare the co-crystalline film of the chip matrix and the sample.

#### Mass Spectrometric Detection and Data Analysis

The prepared chip was loaded onto the MassARRAY Analyzer four system for sample detection. Typer 4.0 software was used to obtain the original data and construct a cluster diagram for bioinformatics analysis.

#### Statistical Analysis

Data were input into Microsoft Excel and sorted and were then analyzed using SPSS 20.0 statistical software (IBM, Armonk, NY, United States). Data that were normally distributed are expressed as the mean ± standard deviation and were analyzed by analysis of variance. Data that did not conform to a normal distribution are expressed as the median (upper quartile, lower quartile) and were analyzed using nonparametric statistical methods. Categorical data were analyzed by the chi-square test. Hardy–Weinberg equilibrium was assessed by the chi-square test using SPSS 20.0, and the genotype and allele frequencies were compared between the schizophrenia and the control groups. Pairwise SNP linkage disequilibrium analysis and association analyses of SNPs and haplotypes were performed using the online software SHEsis (http://analysis.bio-x.cn/SHEsisMain.htm/). Odds ratios (OR) and 95% confidence intervals (95% Cls) were obtained.

Linkage disequilibrium between paired SNPs was analyzed, and the degree of linkage disequilibrium between each pair of SNPs was expressed as D′. The value of D′ ranges from 0 to 1, with a higher value indicating a higher degree of linkage disequilibrium between the two loci. Polymorphic loci were grouped according to their D′ values, and SNPs with a high degree of linkage disequilibrium were combined into groups for haplotype analysis using the “Pair-loci D'/r2 value” option in SHEsis.

The “Haplotype analysis” option in SHEsis was used to analyze haplotypes. The haplotype frequency was estimated using the expectation-maximization method, and the threshold value was set at 0.03. Taking the allelic genomic combination with the highest frequency as a reference, the ORs and 95% Cls of other allelic combinations were calculated. The overall *p* value was used to determine whether the haplotype was associated with schizophrenia, using *p* < 0.05 to indicate a significant difference. The results showed that the allelic combination with the highest frequency was the haplotype associated with schizophrenia. Power analysis was performed using G*Power 3.1 software.

## Results

### Comparison of General Demographic Data Between the two Groups

In the schizophrenia group, there were 92 males and 42 females, aged from 16 to 58 years. The duration of education, age of onset, total PANSS score, positive symptom score, negative symptom score, and general psychotic symptom score were 9.17 ± 2.33 years, 23.78 ± 7.60 years, 90.83 ± 29.85, 13.45 ± 5.98, 17.66 ± 8.55, and 28.80 ± 8.71, respectively. There were 64 individuals in the control group, including 38 males and 26 females, aged from 22 to 59 years, with an average age of 45.6 ± 5.47 years. The duration of education in the control group was 10.62 ± 4.58 years. There was no significant difference in the male:female ratio between the two groups (*p* > 0.05), but there were significant differences in age and the duration of education between the two groups (*p* < 0.05).

### Results of Genomic DNA Extraction

The OD values of the extracted genomic DNA showed DNA concentrations greater than 20 ng/μl, with OD 260 nm/280 nm ratios between 1.6 and 2.2 and OD 260 nm/230 nm ratios greater than 0.6230. There was no absorption peak and the DNA bands were intact, without serious degradation, which met the requirements for subsequent SNP analysis. The gel electrophoresis results of the DNA samples are shown in Supplementary Figure S1.

### Test of Hardy-Weinberg Equilibrium

Genotype data for the rs11030101, rs2030324, and rs6265 polymorphisms in the *BDNF* gene and the rs6740584 and rs2551640 polymorphisms in the *CREB* gene for 134 patients with schizophrenia were tested for Hardy–Weinberg equilibrium. There were no significant differences between the observed genotype frequencies at these five loci and their expected population frequencies, as shown in Tables 1, 2 (*p* > 0.05).

**TABLE 1:** Hardy–Weinberg equilibrium test for *BDNF* genotypes.

Locus	Group (*n*)	Genotype	Frequency (%)	X^2^	*p*
rs11030101	schizophrenia group (134)	AA	79 (58.9)	0.42	0.81
		AT	46 (34.3)	—	—
		TT	9 (6.7)	—	—
	control group (64)	AA	40 (62.5)	0.24	0.88
		AT	22 (34.4)	—	—
		TT	2 (3.1)	—	—
rs2030324	schizophrenia group (134)	AA	44 (32.8)	0.08	0.96
		AG	67 (50.0)	—	—
		GG	23 (17.2)	—	—
	control group (64)	AA	21 (32.8)	1.27	0.53
		AG	35 (54.7)	—	—
		GG	8 (12.5)	—	—
rs6265	schizophrenia group (134)	CC	37 (27.6)	0.93	0.63
		CT	72 (53.7)	—	—
		TT	25 (18.7)	—	—
	control group (64)	CC	12 (18.8)	0.33	0.85
		CT	34 (53.1)	—	—
		TT	18 (28.1)	—	—

**TABLE 2:** Hardy–Weinberg equilibrium test for *CREB* genotypes.

Locus	Group (*n*)	Genotype	Frequency (%)	X^2^	*p*
rs6740584	schizophrenia group (134)	CC	56 (41.8)	0.42	0.81
		CT	58 (43.3)	—	—
		TT	20 (14.9)	—	—
	control group (64)	CC	31 (48.4)	0.15	0.93
		CT	28 (43.8)	—	—
		TT	5 (7.8)	—	—
rs2551640	schizophrenia group (134)	AA	52 (38.8)	0.00	1.00
		AG	63 (47.0)	—	—
		GG	19 (14.2)	—	—
	control group (64)	AA	29 (45.3)	0.53	0.77
		AG	30 (46.9)	—	—
		GG	5 (7.8)	—	—

The distribution of genotype frequencies at each locus showed that the genotype at the *BDNF* locus, rs11030101, was predominantly AA in both the schizophrenia and control groups; the rs2030327 genotype was predominantly AG; and the rs6265 genotype was predominantly CT. The *CREB* genotype at the rs6740584 locus was predominantly CT (43.3%) in the schizophrenia group and CC (48.4%) in the control group, whereas the genotype at the rs2551640 locus was predominantly AG in both the schizophrenia and control groups.

### Results of Genotype Analysis

As shown in Tables 3, 4, there were no significant differences in genotype frequencies at the *BDNF* gene loci rs11030101, rs2030324, or rs6265 between the two groups (*p* > 0.05). Genotype frequencies at the *CREB* gene loci rs6740584 and rs2551640 also showed no significant differences between the two groups (*p* > 0.05). Moreover, there were no significant differences in these genotype frequencies according to gender (*p* > 0.05).

**TABLE 3:** Comparison of *BDNF* rs11030101, rs2030324, and rs6265 genotype frequencies between the two groups.

Group	Gender	Cases(*n*)	rs11030101			rs2030324			rs6265		
			AA	At	TT	AA	AG	Gg	CC	CT	TT
schizophrenia group	Male	92	55	29	8	29	43	20	25	51	16
control group	Male	38	25	12	1	13	19	6	8	19	11
schizophrenia group	female	42	24	17	1	15	24	3	12	21	9
control group	female	26	15	10	1	8	16	2	4	15	7
*p*	male	—	0.452	0.742	0.321	—	—	—	—	—	—
*p*	female	—	0.935	0.916	0.456	—	—	—	—	—	—
*p*	*—*	*—*	0.578	0.675	0.205	—	—	—	—	—	—

**TABLE 4:** Comparison of *CREB* rs6740584 and rs2551640 genotype frequencies between the two groups.

Group	Gender	Cases(*n*)	rs6740584			rs2551640		
			CC	CT	TT	AA	AG	Gg
schizophrenia group	Male	92	37	44	11	34	48	10
control group	male	38	19	18	1	19	18	1
schizophrenia group	female	42	19	14	9	18	15	9
control group	female	26	12	10	4	10	12	4
*p*	male	—	0.209	0.183	—	—	—	—
*p*	female	—	0.807	0.663	—	—	—	—
*p*	*—*	*—*	0.337	0.217	—	—	—	—

### Results of Allelic Analysis

As shown in Table 5, there were no significant differences in rs11030101, rs2030324, rs6265, rs6740584, or rs2551640 allele frequencies between the two groups (*p* > 0.05), and there were no significant differences in allele frequencies according to Gender.

**TABLE 5:** Comparison of rs11030101, rs2030324, rs6265, rs6740584, and rs2551640 allele frequencies between the two groups.

Group	Gender	Cases(*n*)	rs11030101		rs2030324		rs6265		rs6740584		rs2551640	
			A	T	A	G	C	T	C	T	A	G
schizophrenia group	male	92	139	45	101	83	101	83	118	66	116	68
control group	male	38	62	14	45	31	35	41	56	20	56	20
schizophrenia group	female	42	65	19	54	30	45	39	52	32	41	33
control group	female	26	40	12	32	20	23	29	34	18	32	20
*p*	male	—	0.331	0.523	0.194	0.136	0.099	—	—	—	—	—
*p*	female	—	0.951	0.747	0.290	0.683	0.492	—	—	—	—	—
*p*	*—*	*—*	0.428	0.661	0.088	0.178	0.129	—	—	—	—	—

### Distribution Analysis of the Genetic Model

As shown in Table 6, there were no significant differences between the patient and control groups in the dominant, recessive, or over-dominant models of the three *BDNF* gene loci, rs11030101, rs2030324, and rs6265, or the two *CREB* gene loci, rs6740584 and rs2551640 (*p* > 0.05).

**TABLE 6:** Genetic model distribution between two groups (n).

Locus	Model	Genotype	Schizophrenia group (n)	Control group (n)	X^2^	*p*	Or (95% CI)
rs11030101	dominant	AA + AT	125	62	1.065	0.302	0.448 (0.094,2.137)
		TT	9	2	—	—	—
	recessive	AA	79	40	0.227	0.634	0.862 (0.467,1.589)
		AT + TT	55	24	—	—	—
	over-dominant	AA + TT	88	42	0.00	0.995	1.002 (0.535,1.876)
		AT	46	22	—	—	—
rs2030324	dominant	AA + AG	111	56	0.714	0.398	0.689 (0.290,1.639)
		GG	23	8	—	—	—
	recessive	AA	44	21	0.00	0.997	1.001 (0.531,1.888)
		AG + GG	90	43	—	—	—
	over-dominant	AA + GG	67	29	0.381	0.537	1.207 (0.664,2.193)
		AG	67	35	—	—	—
rs6265	dominant	TT + CT	97	52	1.827	0.177	0.605 (0.291,1.259)
		CC	37	12	—	—	—
	recessive	TT	25	18	2.284	0.131	0.586 (0.292,1.177)
		CC + CT	109	46	—	—	—
	over-dominant	CC + TT	62	30	0.006	0.936	0.976 (0.537,1.773)
		CT	72	34	—	—	—
rs6740584	Dominant	CC + CT	114	59	1.986	0.159	0.483 (0.173,1.352)
		TT	20	5	—	—	—
	Recessive	CC	56	31	0.777	0.378	0.764 (0.420,1.390)
		CT + TT	78	33	—	—	—
	over-dominant	CC + TT	76	36	0.004	0.951	1.019 (0.559,1.858)
		CT	58	28	—	—	—
rs2551640	Dominant	AA + AG	115	59	1.648	0.199	0.513 (0.182,1.442)
		GG	19	5	—	—	—
	Recessive	AA	52	29	0.759	0.384	0.765 (0.419,1.398)
		AG + GG	82	35	—	—	—
	over-dominant	AA + GG	71	34	0.000	0.985	0.994 (0.548,1.806)
		AG	63	30	—	—	—

### Linkage Disequilibrium Analysis

Linkage disequilibrium (i.e., allelic association) between two loci is generally indicated by a D′ value >0.8. Because of the large distance between the three polymorphic loci of the *BDNF* gene and the two polymorphic loci of the *CREB* gene, the estimated r^2^ values were relatively low. Therefore, the D′ value was used to determine the degree of linkage disequilibrium between loci. Strong linkage disequilibrium was observed between rs11030101, rs2030324, and rs6265, as shown in Table 7. The D′ value for rs6740584 and rs2551640 was 0.953, suggesting strong linkage disequilibrium between these two polymorphic loci of the *CREB* gene.

**TABLE 7:** Results of linkage disequilibrium analysis.

Locus	rs2030324	rs6265
rs11030101	0.975	1.000
rs2030324	—	0.943

### Analysis of the Association Between *BDNF* and *CREB* Haplotypes and Schizophrenia

We analyzed the distribution of *BDNF* (rs11030101, rs2030324, and rs6265) and *CREB* gene (rs6740584 and rs2551640) haplotypes in the schizophrenia and control groups. When constructing the haplotypes for analysis, only those with a frequency of at least 3% were selected to explore their association with schizophrenia. There was a significant difference in the *BDNF* gene rs11030101/rs2030324/rs6265 AAC haplotype frequency between the schizophrenia and control groups (*p* < 0.05, Table 8).

**TABLE 8:** Analysis of haplotype distribution between the schizophrenia and control groups.

Gene	Haplotype	Cases, *n* (frequency)	Controls, *n* (frequency)	*p*	Or (95%CI)
BDNF	AAC	35 (0.132)	8 (0.063)	0.037^a^	2.288 (1.032,5.072)
	AAT	118 (0.442)	69 (0.538)	0.084	0.688 (0.450,1.053)
	AGC	49 (0.181)	26 (0.203)	0.645	0.883 (0.519,1.503)
	TGC	61 (0.227)	24 (0.187)	0.347	1.288 (0.759,2.185)
CREB	CA	163 (0.608)	88 (0.688)	0.215	0.749 (0.474,1.184)
	TG	94 (0.350)	38 (0.297)	0.215	1.335 (0.845,2.109)

a *p* < 0.05.

### Analysis of the Association Between Genotypes and Clinical Symptoms of Schizophrenia

The associations between *BDNF* (rs11030101, rs2030324, and rs6265) and *CREB* genotypes (rs6740584 and rs2551640) and clinical psychiatric symptoms in patients with schizophrenia are shown in Table 9. There were significant differences in the PANSS negative symptom scores, N2 (emotional withdrawal), N3 (communication disorder), N6 (lack of spontaneity and fluency of conversation), and N7 (stereotyped thinking) and total negative symptom scores in patients with different *BDNF* gene rs11030101 genotypes. Moreover, there were significant differences in the PANSS general symptom score G12 (judgment and lack of insight) in patients with different *BDNF* gene rs6265 genotypes (*p* < 0.05).

**TABLE 9:** Relationships between different *BDNF* and *CREB* genotypes and PANSS scores in the schizophrenia group *p* < 0.05.

PANSS	rs11030101		rs2030324		rs6265		rs6740584		rs2551640	
Items	X^2^	p	X^2^	p	Z	p	X^2^	p	X^2^	p
P1	0.782	0.676	5.192	0.075	2.909	0.233	1.724	0.422	2.021	0.364
P2	4.175	0.124	0.904	0.636	1.655	0.437	0.435	0.804	0.019	0.991
P3	0.977	0.613	2.561	0.278	2.142	0.343	1.695	0.428	2.997	0.223
P4	3.944	0.139	1.332	0.514	1.343	0.511	1.027	0.598	1.747	0.417
P5	0.963	0.618	0.154	0.926	0.585	0.747	1.323	0.516	2.265	0.322
P6	1.894	0.388	4.259	0.119	5.380	0.068	2.048	0.359	2.921	0.232
P7	5.423	0.066	1.990	0.370	3.742	0.154	0.269	0.874	0.927	0.629
*p* (total)	0.084	0.959	2.941	0.230	2.443	0.295	1.468	0.480	1.314	0.518
N1	3.275	0.194	0.330	0.848	0.208	0.901	0.541	0.763	3.272	0.195
N2	6.774	0.034*	0.404	0.817	1.089	0.580	0.110	0.946	2.024	0.363
N3	8.390	0.015*	2.118	0.347	1.818	0.403	0.114	0.944	0.788	0.674
N4	5.874	0.053	1.106	0.575	0.412	0.814	1.097	0.578	6.989	0.030
N5	5.620	0.060	0.259	0.878	0.057	0.972	0.344	0.842	0.345	0.841
N6	9.773	0.008*	1.582	0.453	0.018	0.991	0.653	0.722	0.184	0.912
N7	6.329	0.042*	0.217	0.897	2.554	0.279	0.732	0.693	3.600	0.165
N (total)	8.257	0.016*	0.470	0.791	0.065	0.968	0.106	0.948	2.076	0.354
G1	4.553	0.103	1.883	0.390	1.325	0.516	1.607	0.448	0.634	0.728
G2	3.943	0.139	1.183	0.554	1.088	0.580	0.617	0.735	0.502	0.778
G3	3.244	0.198	0.175	0.916	3.151	0.207	2.388	0.303	1.709	0.426
G4	5.016	0.081	0.827	0.661	0.490	0.783	0.018	0.991	1.693	0.429
G5	1.505	0.471	0.670	0.715	0.294	0.863	1.154	0.561	1.283	0.527
G6	1.813	0.404	0.055	0.973	0.054	0.974	0.053	0.974	1.206	0.547
G7	0.914	0.633	0.583	0.747	0.211	0.900	1.033	0.597	2.740	0.254
G8	1.586	0.452	1.766	0.414	0.218	0.897	2.225	0.329	0.090	0.956
G9	0.544	0.762	2.322	0.313	1.716	0.424	0.844	0.656	1.576	0.455
G10	4.822	0.090	0.402	0.818	1.580	0.454	4.321	0.115	3.201	0.202
G11	1.632	0.442	0.575	0.750	0.255	0.880	0.588	0.745	1.789	0.409
G12	2.442	0.295	4.556	0.102	9.338	0.009*	2.371	0.306	1.327	0.504
G13	1.620	0.445	1.917	0.383	0.166	0.921	1.463	0.481	1.541	0.463
G14	0.032	0.984	0.370	0.831	1.450	0.484	0.223	0.894	2.811	0.245
G15	3.929	0.140	2.222	0.329	0.334	0.846	2.028	0.363	4.494	0.106
G16	4.462	0.107	1.023	0.600	0.730	0.694	0.344	0.842	0.902	0.637
G (total)	5.164	0.076	1.155	0.561	1.084	0.582	0.180	0.914	1.417	0.492
PANSS (total)	5.173	0.075	0.153	0.926	0.316	0.854	0.205	0.902	0.558	0.757

### Evaluation of Statistical Power

The G*Power program was used to perform the power calculation. The size of the sample used in this study had a power of 92.372% to detect a significant association (α < 0.05) with a given effect size index value of 0.5.

## Discussion

The findings of the current study can be represented by the following two aspects. First, we found a significant increase in the rs11030101/rs2030324/rs6265 AAC haplotype frequency in schizophrenic subjects compared with controls. Second, genotypes at the rs11030101 and rs6265 loci of the *BDNF* gene were associated with either negative or clinical pathological symptoms, which suggested that *BDNF* gene polymorphisms may be associated with negative symptoms in schizophrenic subjects in southern China.

The neurodevelopmental hypothesis is currently the main etiological explanation of schizophrenia. Under this hypothesis, schizophrenia is thought to be a consequence of disorders in the development and maturation of neurons and neural pathways in the embryonic brain. Meanwhile, the symptoms of schizophrenia are induced by an abnormal environment of the outside world (Zhao and Shi, 2015). In this study, 134 schizophrenic patients and 64 healthy controls were recruited from the Han population in Guangdong Province, China. The allele and genotype frequencies of three SNPs (rs11030101, rs2030324, and rs6265) in the *BDNF* gene and two SNPs in the *CREB* gene (rs6740584 and rs2551640) were compared between the schizophrenia and control groups. During the past 2 decades, many small- and large-scale studies have explored the association between *BDNF* gene polymorphisms (rs11030101, rs2030324, and rs6265) and schizophrenia, mainly with negative results. Moreover, *BDNF* is not included in the list of index schizophrenia loci from the Psychiatric Genomics Consortium’s genome-wide association study of tens of thousands of cases and controls (Pardiñas et al., 2018). In the current study, the distribution of *BDNF* SNP genotypes (rs11030101, rs2030324, and rs6265) was not different between healthy controls and patients with schizophrenia, which is in accordance with the results of previous studies (Zhang et al., 2016b).

A haplotype refers to a random set of multiple alleles at closely linked loci on a given chromosome. The effectiveness of genetic analysis of a single genetic marker is limited. Haplotype analysis of multiple loci makes effective use of genetic information at each locus and increases the testing power. Specific haplotypes containing disease susceptibility genes or resistance genes can be identified using this analysis method, and multiple susceptibility or resistance loci can be identified by analyzing the composition and frequency of haplotypes composed of different SNPs on the same chromosome. Our haplotype analysis showed that the *BDNF* gene rs11030101/rs2030324/rs6265 AAC haplotype was more common in the schizophrenia group than in the control group, suggesting that this haplotype may be related to an increased susceptibility to schizophrenia.

PANSS is one of the most commonly used scales to evaluate the clinical symptoms of patients with schizophrenia. It includes 33 items assessing the positive, negative, and general symptoms of schizophrenia (Kay et al., 1987; He and Zhang, 1997). We found differences in negative scale scores in schizophrenic patients with different *BDNF* genotypes at the rs11030101 locus. Moreover, different genotypes had an effect on the PANSS negative symptom scores, with the AA genotype showing the greatest effect and the TT genotype showing the smallest effect. Clinical negative symptoms were more prominent in schizophrenic patients with the AA genotype at rs11030101. Genetic theory states that if multiple genes are associated with a disease, one of them is associated with a dominant phenotype of the disease. Previous studies (Li et al., 2013; Zhai et al., 2013) have shown that there is a significant association between *BDNF* gene polymorphisms and clinical negative symptoms of schizophrenia. The same results were obtained in this study, suggesting that the rs11030101 locus of the *BDNF* gene plays an important role in the occurrence of clinical negative symptoms of schizophrenia, which provides some clues for clinical diagnosis.

CREB is involved in the intersection of several intracellular signal transduction pathways. In fact, the cyclic adenosine monophosphate, mitogen-activated protein kinase, calcium-dependent protein kinase, and glycogen synthase kinase three pathways form four upstream pathways of CREB. These four pathways eventually regulate the expression of *BDNF* and other downstream genes, thus affecting neuronal plasticity and neurotransmitter synthesis (Lu et al., 2008). As a nuclear regulatory factor in eukaryotes, CREB plays an important role in neuronal regeneration, synaptic plasticity, learning, and memory. CREB is thought to be involved in the differentiation, survival, and migration of early hippocampal progenitor cells (Kandel, 2012). In 1999, Kawanishi et al. (1999) found that schizophrenic patients with the C allele at the *CREB* gene T933TC polymorphism had general clinical manifestations and certain unique symptoms. We found no significant differences in rs6740584 or rs2551640 genotype or allele frequencies between schizophrenic patients and healthy control subjects. Moreover, no positive associations were found in genetic model, haplotype, and symptom analyses, which was consistent with the results reported by Forero et al. (2016) and Bai Lijuan et al. (Bai et al., 2016). To some extent, this study showed that *CREB* gene polymorphisms are not related to susceptibility to schizophrenia. In addition, these findings indicate that other factors may be involved in the pathogenesis of schizophrenia. Generally speaking, as an important susceptibility gene for fever in schizophrenic patients, polymorphisms of the *BDNF* gene may be related to the susceptibility to schizophrenia and the severity of symptoms.

The statistical power of this study was evaluated using G*Power 3.1 software. The total sample size used for the allele, genotype, and haplotype frequency analyses gave a statistical power of 92.372% for α < 0.05. Thus, although the sample size was small, the total sample had good statistical validity and the results were statistically significant. Similarly, Kumar et al. (2020) obtained significant results with a small sample size. They found that the serum BDNF concentration was significantly lower in schizophrenic patients than healthy individuals, and that the rs6265 polymorphism was not associated with schizophrenia in a case-control study of 50 schizophrenic patients and 50 healthy individuals.

A major limitation of this study was the small sample size, which translates to an underpowered study in terms of *BDNF* (rs11030101, rs2030324, and rs6265) and *CREB* (rs6740584 and rs2551640) genotype distributions. This may be considered a serious methodological limitation that prevents definitive conclusions about the role of the *BDNF* gene in schizophrenia in the Chinese Han population. In the future, more large-scale studies in different populations are needed to verify the reliability of the association and contribute to a deeper understanding of the etiology and mechanism of schizophrenia. This study provides the basis for revealing the genetic mechanisms of susceptibility to schizophrenia and the severity of the associated symptoms.

## Data Availability

The original contributions presented in the study are included in the article/Supplementary Material. Further inquiries can be directly to the corresponding authors.
